# Anxiety and Depression as Predictive Factors of Quality of Life in Old Age: A Longitudinal Analysis

**DOI:** 10.1093/geroni/igaa057.3315

**Published:** 2020-12-16

**Authors:** Oscar Ribeiro, Lia Araújo, Laetitia Teixeira, Carmen Rodríguez-Blázquez, Maria Joao Forjaz

**Affiliations:** 1 University of Aveiro, Aveiro, Portugal; 2 Instituto Politécnico de Viseu, Viseu, Portugal; 3 ^3^ Institute of Biomedical Sciences Abel Salazar | Center for Health Technology and Services Research (CINTESIS), University of Porto, Porto, Portugal; 4 Carlos III Health Institute, Madrid, Spain

## Abstract

Research on the effect of age in quality of life (QoL) is controversial, and doubts remain if this effect is continuous and if it disappears when controlling for other factors. Most studies have verified the association of health-related factors, such as poor physical health, functional impairment, and depression with lower scores of QoL in old age. But studies that analyse such an association in non-clinical samples through longitudinal analysis and including anxiety as a mental health factor remain very rare. This study relies on data from two waves from the Survey of Health, Ageing and Retirement in Europe (SHARE) and focuses on a representative sample of individuals aged 50+ from Portugal. It comprises 1782 participants (baseline), of which 1201 were reassessed. The mean age was 64.8 years (sd=9.3 years) and 55.4% were female. Linear Mixed Effects models were performed to identify potential factors associated with changes of QoL across age. According with the final model (pseudo-R2 equal to 0.421), a significant effect of age as time-dependent factor was verified, observing a decline of QoL with increasing age. Years of education, depression and anxiety at baseline were significantly associated with QoL. Thus, the effect of age on quality of life remains significant even when other factors are considered. The influence of mental health on QoL reinforces the importance of intervening in both depressive and anxiety symptoms in old age.

